# Magnetic Resonance Imaging and Modeling of the Glymphatic System

**DOI:** 10.3390/diagnostics10060344

**Published:** 2020-05-27

**Authors:** Jasleen Kaur, Esmaeil Davoodi-Bojd, Lara M Fahmy, Li Zhang, Guangliang Ding, Jiani Hu, Zhenggang Zhang, Michael Chopp, Quan Jiang

**Affiliations:** 1 Department of Neurology, Henry Ford Health System, Detroit, MI 48202, USA; jasleenkaur@oakland.edu (J.K.); EDAVOOD1@hfhs.org (E.D.-B.); larafahmy@wayne.edu (L.M.F.); lzhang3@hfhs.org (L.Z.); gding1@hfhs.org (G.D.); ZZHANG1@hfhs.org (Z.Z.); MCHOPP1@hfhs.org (M.C.); 2 Department of Physics, Oakland University, Rochester, MI 48309, USA; 3 Department of Radiology, Henry Ford Health System, Detroit, MI 48202, USA; 4 Department of Psychiatry and Behavioral Neurosciences, Wayne State University, Detroit, MI 48201, USA; 5 Department of Radiology, Wayne State University, Detroit, MI 48201, USA; jhu@med.wayne.edu

**Keywords:** glymphatic system, contrast-enhanced MRI, cerebrospinal fluid, computational modeling, aquaporin-4, flow pathways, extracellular space, neurological diseases, bulk flow, diffusion

## Abstract

The glymphatic system is a newly discovered waste drainage pathway in the brain; it plays an important role in many neurological diseases. Ongoing research utilizing various cerebrospinal fluid tracer infusions, either directly or indirectly into the brain parenchyma, is investigating clearance pathways by using distinct imaging techniques. In the present review, we discuss the role of the glymphatic system in various neurological diseases and efflux pathways of brain waste clearance based on current evidence and controversies. We mainly focus on new magnetic resonance imaging (MRI) modeling techniques, along with traditional computational modeling, for a better understanding of the glymphatic system function. Future sophisticated modeling techniques hold the potential to generate quantitative maps for glymphatic system parameters that could contribute to the diagnosis, monitoring, and prognosis of neurological diseases. The non-invasive nature of MRI may provide a safe and effective way to translate glymphatic system measurements from bench-to-bedside.

## 1. Introduction

**Figure 1:**
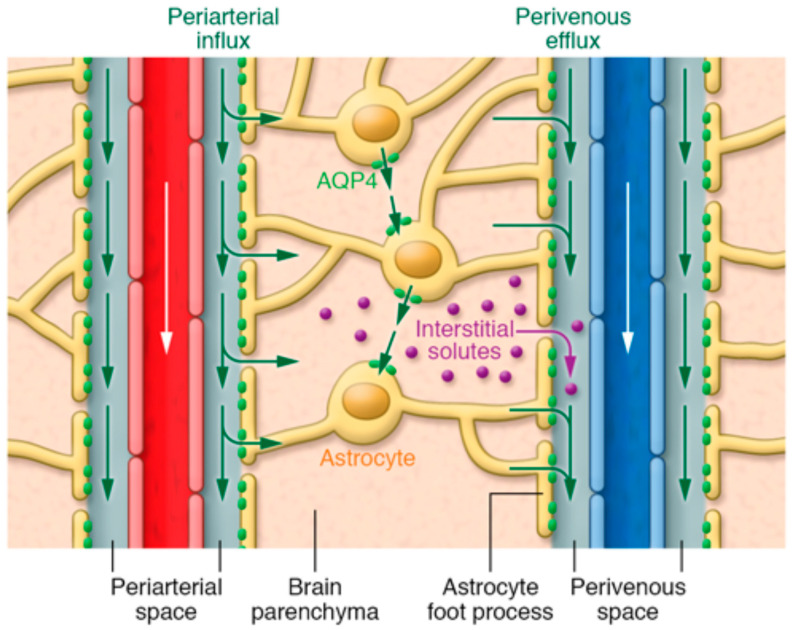
Illustration of fluid and interstitial waste solutes movement in the brain between the parenchyma and perivascular spaces surrounding the penetrating vasculature. The periarterial influx in the figure shows the inward movement of CSF along the periarterial space, which mixes with the ISF and interstitial waste solutes in the parenchyma with the help of AQP-4 and gaps between astrocytic end-feet, and then exit via perivenous efflux pathway (along the perivenous space). White arrows indicate the direction of blood flow in the vasculature. Green arrows show the CSF and CSF-ISF fluid transport (whether by diffusion, bulk flow, or both remains elusive). Purple dots indicate the interstitial waste solutes that exit the parenchyma through gaps between the astrocytic end-feet into the perivenous space, which are then drained to the CSF. Reproduced with permission from [15].

According to the traditional understanding of cerebrospinal fluid (CSF) dynamics, CSF (which is mainly produced by the choroid plexus at a rate of 0.3–0.4 mL/min) passes from the lateral ventricles to the third ventricle through the interventricular foramen of Monro and then goes to the fourth ventricle through the cerebral aqueduct of Sylvius [1,2]. From the fourth ventricle, CSF makes its way into the subarachnoid space via the median aperture (foramen of Magendie) and the two lateral apertures (foramina of Luschka) [2,3]. The metabolic waste from the parenchyma mixes with the CSF in the subarachnoid space and exits via the spinal and cranial nerves, as well as the arachnoid villi [4]. Recent studies have expanded upon this traditional understanding of CSF dynamics, demonstrating the presence of CSF within the brain parenchyma (termed the glymphatic system) [5,6,7,8,9,10]. The glymphatic system assists in the transport of glucose, lipids, signaling molecules, and apolipoprotein-E into the brain parenchyma [7,11,12], and it promotes the clearance of proteins and interstitial waste solutes out of the brain to maintain brain homeostasis [5,6,13,14]. Approximately eight years ago, Dr. Nedergaard’s group from the University of Rochester identified the glymphatic system in rodents [5]. They proposed that a significant portion of the subarachnoid CSF enters the brain parenchyma via peri-arterial spaces and then mixes with the interstitial fluid (ISF) and the waste solutes produced by cellular metabolism. The resulting CSF-ISF fluid exchange and the interstitial waste solutes then exit along explicit peri-venous spaces alongside parenchymal venous blood vessels [5,6,13,14], as demonstrated in Figure 1 [15]. Aquaporin-4 water channel proteins (AQP-4), embedded in the astrocytic end-feet, along with astrocytic end-feet gap junctions, have been proposed to play a critical role in the glymphatic system [5,16,17]; interstitial waste solute clearance declines by ~70% in transgenic mice lacking AQP-4 compared to wild type mice [5]. Ultra-fast magnetic resonance encephalography in human brain studies has suggested that three distinct pulsation mechanisms, cardiac (0.8–1.2 Hz), respiratory (0.2–0.3 Hz), and very low frequency (<0.1 Hz) pulsations, are together responsible for the flow of CSF and the efflux of protein waste solutes from the brain [18]. Arterial pulsations, particularly cardiac systole, are thought to be the major contributor to the CSF bulk flow and CSF-ISF fluid exchange through AQP-4, thus providing solute transport from peri-arterial spaces into the extracellular brain tissue [14,19,20]. 

Normal physiology (such as blood pressure [21], sleep [14], anesthesia [22], body posture [23], and aging [19,21,24,25,26,27,28,29,30]) affects the glymphatic system. It was demonstrated that a 10 bpm decrease in heart rate (bradycardia) reduces waste clearance and results in a nearly 20% additional accumulation of beta-amyloid in the brain parenchyma, whereas a 30 bpm increase in heart rate (tachycardia) showed a nearly 30% reduction in beta-amyloid levels in the brain parenchyma [21]. Additionally, the glymphatic system in mice is most dynamic during sleep and removes beta-amyloid with exceptional efficiency, with a more than two-fold increase in clearance rate [14]. Anesthetics also affect the glymphatic system, and a nearly 30% increase in solute transport can be seen in rats that are anesthetized with dexmedetomidine plus low-dose isoflurane, as compared to high-dose isoflurane only [22]. Results from contrast-enhanced magnetic resonance imaging (MRI) in rats validated by quantitative optical imaging in mice demonstrated the role of body posture/positioning on the glymphatic system, thus indicating that the right lateral position is the most efficient for waste clearance as compared to supine and prone positions [23]. During aging, ventricular shrinkage and significant reductions in CSF production and pressure, along with decreased arterial pulsatility (due to arterial stiffening), lead to significant reductions in glymphatic system function (~80–90% reduction in old mice compared to young mice), thus leading to a critical increment in beta-amyloid deposition within the brain parenchyma [19,21,24,25,26,27,28]. Aged mice also exhibited an impairment of the meningeal lymphatic function, which slows down the clearance of CSF macromolecules from the brain and could lead to Alzheimer’s disease [29].

Impairments in the glymphatic system are associated with several neurological diseases. These diseases include but are not limited to Alzheimer’s disease (AD) [31,32,33], small vessel disease (SVD) [34,35], diabetes [36], traumatic brain injury (TBI) [9,24,37,38,39,40,41], and stroke [42,43,44]. In many instances, it is unclear whether impairments in the glymphatic system are the cause or the effect (or both) of these diseases [34]. AD has been proposed to be caused by the accumulation of tau proteins and beta-amyloid protein waste deposition, which cluster in the form of plaques inside the brain, causing neurotoxicity and memory degradation. Impairment of glymphatic system could be one major cause of these waste deposition in AD [31,32,33]. In SVD [45], damage to the small end arteries, arterioles, capillaries, and venules, along with the expansion of the perivascular spaces (particularly in older individuals), adversely affects the glymphatic system function and thus evokes white and grey matter damage that can ultimately lead to dementia [34,35,46]. TBI induces axonal damage, which is responsible for the abnormal discharge of tau proteins into the ISF and CSF of the brain, thus significantly impacting glymphatic system function [9,24,37,38,39,40,41]. Acute ischemic stroke and subarachnoid hemorrhage (SAH) substantially impair the functioning of the glymphatic system [42,43,44]. A very recent human study with acute ischemic stroke patients who encountered intraprocedural extravasation during thrombectomy demonstrated the possible evidence of the glymphatic clearance of iodine contrast using serial computed tomography imaging [47]. Diabetes has been proposed to play a role in triggering cognitive impairment/dysfunction [48,49,50], but the exact mechanism of such is not completely understood. Our previous study showed that type-2 diabetes mellitus (DM) in rats is responsible for a reduction in interstitial waste solutes clearance from the brain and could induce other cognitive deficits [36]. Further investigations of neurological diseases and their interactions with the glymphatic system may lead to new approaches to reduce neurological deficits and to sustain healthy aging. Moreover, by investigating these neurological diseases with various imaging techniques, particularly prior to clinical symptom onset, we can potentially identify a therapeutic window for treatment.

## 2. Efflux Pathways for Brain Waste Clearance

The influx pathways of the glymphatic system through peri-arterial spaces are relatively well-established [5,6,22,23,51]; however, the efflux pathways remain largely understudied and highly debated, perhaps due to technical difficulties. According to the glymphatic pathway, CSF mixed with interstitial waste solutes drain into the perivenous spaces [5] and then goes to the lymphatic system [52]. Though there seems to be a consensus pertaining to the involvement of vascular basement membranes in drainage pathways [5,53,54], there seems to be a debate concerning whether waste solutes drain upstream along the arteries or downstream along veins [55]. Regardless of whether the peri-venous vs the peri-arterial routes are utilized for interstitial waste solutes efflux from the brain parenchyma, three distinct pathways for brain waste clearance out of the central nervous system (CNS) have been proposed [52]: (1) arachnoid granulations; (2) meningeal lymphatics; (3) nasal lymphatics and cranial and spinal nerves. In the first pathway, CSF from the subarachnoid space straightforwardly drains through arachnoid villi of the dural venous sinus into the blood [4,52,56,57,58]; however, this route is controversial because it was proposed to be dominant only in late fetal and early neonatal periods [4,59,60,61] and it loses its efficiency with aging due to the degeneration of arachnoid villi and the thickening of the arachnoid membrane [4]. Experiments in monkeys showed that the arachnoid villi contain very complex channels and are only open for the waste solutes to pass through when the CSF pressure is sufficiently greater than the pressure in the dural venous sinus [62]; moreover, there is a size limitation for the solutes. The diameter of arachnoid villi has been proposed to be 4–12 μm (estimated), but the size of the solutes that can pass through do not only depend on the threshold value of the diameter of up to 7.5 μm, as it also depends on various characteristics such as the consistency, shape, and surface properties of the particles [4,62]. In the second pathway, CSF enters into the meningeal lymphatic vessels that eventually drain into the deep cervical lymph nodes [52,63,64,65]. Recently, Louveau et al. [64,65] and Aspelund et al. [63] described the role of meningeal lymphatic vessels, which are present within the dura mater (mirroring the vasculature), in the drainage of ISF–CSF and waste solutes into the deep cervical lymph nodes in mice. The presence of meningeal lymphatic vessels in human and non-human primates have also been demonstrated by high-resolution clinical MRI studies [66] and confocal microscopy [67]. In the third pathway, CSF enters into subarachnoid spaces around the cranial and spinal nerves (perineurally) and the nasal lymphatics, which also drain into the cervical lymph nodes and thereafter eventually join the systemic blood circulation [4,52,55,68,69,70,71,72,73,74,75,76,77,78].

## 3. Magnetic Resonance Imaging (MRI) Detection and Modeling

The utility of MRI in investigating the glymphatic system has recently gained momentum. Though optical imaging techniques such as two-photon microscopy have classically dominated the field, due to its excellent spatial resolution that is needed to capture small perivascular spaces, its limitations have pushed investigators towards MRI; MRI overcomes the low-penetration depth of two-photon microscopy and allows for whole-brain imaging unlike two-photon microscopy, which is only favorable in imaging small areas of the brain cortex. Beyond the use of contrast agents, image contrast in MRI can be easily altered by the elements of the imaging sequences and parameters, unlike two-photon microscopy. Moreover, different MRI methodologies can capture different elements of the glymphatic system. For example, in vivo, perivascular fluid movement in the glymphatic system and its diffusion properties can be assessed with magnetic resonance diffusion tensor imaging (DTI) [79,80]. MRI is also minimally invasive and therefore can be used to study the glymphatic system in vivo with minimum disruption in both animals and humans. Recent examinations of the human brain that utilized MRI provided evidence that suggested that the presence of the glymphatic system in the human brain is analogous to that found in the rodent brain [81,82,83,84,85,86,87].

Modeling the glymphatic system using data from MRI can provide insight into the glymphatic flow pathways. Iliff et al. [6] demonstrated that MRI is an extraordinary tool for studying the glymphatic pathways, providing good spatial and temporal resolution of the whole rat brain. MRI modeling has been used to capture the association between glymphatic system impairments and neurological diseases/conditions. Dynamic contrast-enhanced MRI, along with multiphoton imaging in mice, has shown that stroke profoundly impacts the functioning of the glymphatic system. During acute focal ischemia, a rapid increase in CSF influx driven by ischemic spreading depolarizations and vasoconstriction contributes to brain edema [44]. Moreover, in the case of cerebral microinfarcts, while a decreased glymphatic influx has been observed after microinfarction, persistent solute retention within microinfarcts has been detected in up to 14 days post-microinfarction. Thus, the dysregulation of the glymphatic system may also contribute to the neuropathology of stroke. This study is fundamentally important in changing the mechanism of stroke-induced edema from the vascular to the glymphatic system [44]. Slow waste clearance from the brain after SAH and ischemic stroke was demonstrated in whole brain scans using MRI [42,43]. MRI was utilized to investigate the impairment of the glymphatic system functioning in four stroke models in mice (SAH, embolic ischemic stroke, intracerebral hemorrhage, and carotid ligature); severe impairment of glymphatic system was demonstrated with SAH and acute embolic ischemic stroke, but intracerebral hemorrhage and carotid ligature did not contribute to the glymphatic impairment [42]. Another MRI study in non-human primates showed significant CSF circulation impairment in the brain parenchyma after SAH, which may lead to the dysfunction of the glymphatic system [43]. We have demonstrated a reduction in tracer clearance in rat models of DM compared to age matched controls using MRI [36]. An in vivo MRI analysis of contrast agent gadolinium-diethylenetriamine pentaacetic acid (Gd-DTPA) clearance from interstitial space, also validated by ex vivo fluorescence microscopic images, showed that the clearance rate constant is decreased by three times in the hippocampus of DM rats as compared to non-DM rats [36]. Using cluster analysis, changes in the distribution of the fast, intermediate, and slow glymphatic pathways have been derived. DM has been found to significantly increase the perivascular space (the fast glymphatic pathway), and a large accumulation of solutes in a DM animal may be caused by reduced clearance of intermediate glymphatic pathway [36]. MRI has provided the evidence of presence of meningeal lymphatic vessels in human and non-human primates for CNS waste clearance [66]. A very recent study on humans utilizing MRI provided a clinical methodology to simultaneously visualize the glymphatic system clearance, deep cervical lymph nodes, and putative meningeal lymphatic vessels [30]—this MRI study also reported decreased glymphatic system and meningeal lymphatic CSF tracer clearance with increased age [30].

### 3.1. Bulk Flow and Diffusion

To maintain brain homeostasis, toxic waste products generated in the brain must be evacuated. At first, it was believed that the removal of protein wastes from the brain occurs solely by diffusion, which can be examined utilizing a real-time iontophoresis method [88], and then the waste solutes drain into the regional lymph nodes. Multiple experiments with an injection of radiolabeled tracers have depicted the diffusive nature for the local movement of the tracers through the extracellular spaces [89,90,91]. Using integrative optical imaging (IOI), Tao and Nicholson qualitatively demonstrated that large molecular weight albumin proteins diffuse through extracellular spaces but are more hindered than small molecular weight proteins [92]. Ex vivo electron microscopy studies have shown the width of the brain extracellular spaces to be approximately 10–20 nm [93,94,95,96]. However, it has been subsequently demonstrated that the extracellular spaces in living animals are at least two-fold wider and are estimated to be approximately 38–64 nm; additionally, the transport of quantum dots (35 nm in size) is primarily due to diffusion [97]. Additionally, the extracellular matrix comprising of adhesive/anti-adhesive proteins can vitally impact the diffusion of certain ions into extracellular spaces [98,99,100,101]. Recent studies considering the tortuous extracellular spaces in the brain parenchyma have suggested the transport of waste solutes by a diffusive mechanism, predominantly depending on solute size [102,103]. 

Looking at the rapid removal of metabolic wastes out of the brain, it was argued that diffusion alone cannot be responsible for waste drainage. Cserr et al. proposed the bulk flow of ISF through explicit channels as the dominant mechanism of waste drainage compared to diffusion by infusing 2000 KDa blue dextran [104] and 40 KDa horseradish peroxidase [105] into the caudate nucleus. Likewise, other trials utilizing different tracers of various atomic sizes have proposed the interstitial bulk flow for CSF–ISF exchange as a dominant clearance pathway [105,106]. On the other hand, in favor of diffusion in extracellular spaces [107,108], it has been proposed that high hydraulic resistance from tight extracellular spaces cannot permit bulk flow [109,110,111,112]. More recently, Nedergaard and colleagues demonstrated that the AQP-4-dependent bulk flow of ISF through extracellular spaces is a critical contributor for interstitial waste solutes clearance from brain parenchyma compared to diffusion (using intra-parenchymal injections) in mice [5]. Another analysis by Lori Ray et al. suggested both diffusion and bulk flow as potential mechanisms of the interstitial solute transport depending on the solute size; bulk flow becomes dominant with larger solute size [15]. Contradictory conclusions for the dominant waste clearance mechanism still exist between diffusion and bulk flow [15,107,108,113].

A computational analysis utilizing the effective diffusion due to the convoluted extracellular spaces demonstrated that dispersion, which is a combination of bulk flow and diffusion, mediates the observed [5,54,114,115,116] fast solute transport [117], but perivascular drainage is the principal system [118], and arterial pulsations alone cannot be responsible for the bulk flow; however, those pulsations contribute to the fast transport due to dispersion [117]. The glymphatic system is consequently dependent on dispersion, which is partly reliant on diffusion and partly reliant upon a faster system of bulk flow [109,110,113,115,116,117,118,119,120,121,122].

### 3.2. Quantitative Bio-Physical Modeling

Mathematical modeling utilizing MRI data has been performed by various groups to better understand the dynamics of the glymphatic system flow. In these studies, a mathematical model was developed expressing the dynamics of the nuclear magnetic resonance (NMR) tracer in a contrast-enhanced MRI of rodents. The measured MRI signal was assumed to be proportional to the tracer concentration in the imaging voxels. Therefore, the dynamic changes of the tracer concentration could be measured by consecutive MRI scans. The aim was to develop a mathematical model based on the tracer behavior in the tissues and underlying tissue characteristics to mimic the measured dynamic changes by MRI. Thus, inversely solving these models could provide quantitative maps of the glymphatic dynamic and structure. 

#### 3.2.1. Modeling Using Multi-Compartment Kinetic Framework

Lee et al. [23] introduced a two-compartment kinetic model based on contrast-enhanced MRI data to analyze the movement of a Gd-DTPA tracer (infused into the cisterna magna) for three distinct body postures in rats. The brain was divided into two sections for this experiment (infusion site and all other brain tissues). By comparing the kinetic parameters (retention and loss) for all the three postures, it was concluded that the glymphatic transport is most effective while sleeping in the lateral position and less proficient in the prone and supine positions [23]. The limitation of this model is that the parameters were derived using a global input function from the infusion site instead of using local input functions separately for each region, which could, therefore, produce more errors for the study of the glymphatic system [23]. In cerebral permeability and perfusion measurements, a global input function has long been a major source of inaccuracy, and despite the utilization of modeling techniques to find the local input functions, it has remained a challenge [123,124]. 

However, our recent study presented the local input functions to visualize the more reliable glymphatic flow pathways of the Gd-DTPA tracer in rats using the two-compartment model [125]. This model (1) utilized cluster analysis to group brain voxels into different regions with similar time signal curves of the contrast-enhanced MRI measurements, (2) computed the local input functions based on the response to the contrast agent infusion, and (3) solved the two-compartment kinetic equations for each region [125]. The limitation of this model is the absence of a diffusion term in the equations used for modeling, which may induce errors for waste clearance in the extracellular spaces of the brain.

#### 3.2.2. Modeling Using Optimal Mass Transport Framework

Ratner et al. [126,127] fitted a mathematical model to the dynamic MRI images of rodent brain injected with the Gd-DTPA tracer into the cisterna magna, and they utilized 3D visualization computational tool to visualize the glymphatic flow vector fields. Here, the tracer flow was estimated between each pair of consecutive MRI images using the theory of optimal mass transport (OMT). Some assumptions of this model include that the injected tracer moves along the glymphatic pathway, the total injected mass is conserved, and the image brightness corresponds to the tracer concentration. Glymphatic pathways were presented as color-coded maps of streamlines per voxel. The OMT algorithm, also known as the Monge-Kantorovich problem, qualitatively demonstrated the directionality of the glymphatic system bulk flow, but the measurements do not provide the quantitative values for directionality as well as the efflux pathways out of the brain [126,127].

Elkin et al. analyzed the glymphatic pathways using the OMT algorithm with a Lagrangian framework instead of using the Eulerian framework [128,129]. The Lagrangian approach was advantageous in envisioning the time-varying glymphatic fluid pathways in over 30 min in a single image [128]. Modeling based on dynamic contrast-enhanced MRI data showed different responses in rats under two different anesthetics. In this model, the continuity equation for the contrast agent was modified by augmenting the diffusion term to regularize the fluid flow for continuous pathways in the glymphatic system [128]. 

In another study by Elkin et al. [129], they again used the Lagrangian approach with a modified continuity equation to more precisely model the fluid flow behavior. A regularized optimal transport procedure and a flow pattern analysis (FPA) were used to find the directional information of the tracer between the time points of the initial and final observed density images from contrast-enhanced MRI of normal rat brains. FPA returned the streamlines for each time step in the whole-time domain, and these were then clustered utilizing the QuickBundles algorithm to picture the glymphatic flow pathways. The major advantage of this model is that it reduces the errors by five times when compared to the customary OMT model; however, it still provides qualitative fluid flow information instead of quantitative information [129]. 

In the most recent paper of vascular investigation by Elkin et al., fluid motion was described by applying an OMT regularized model to the human MRI data of ten patients with head and neck squamous cell carcinoma. The advantages of this model are that it provides quantitative information for the fluid flow with and without considering diffusion into account and it provides a clear signal between the neighboring voxels instead of ignoring intervoxel contrast agent movements [130]. This model can be further used to track the glymphatic pathways, thus showing both bulk flow and diffusion values, which are both critical for the complete understanding of the glymphatic system. 

#### 3.2.3. Modeling Efflux Using DTI

Kim et al. [131] used a voxelized model including a diffusion term for the convective-enhanced delivery (CED) of therapeutics for the treatment of various CNS diseases. This model is of special interest because it can be utilized to model the efflux pathways of the glymphatic system. In this model, data from DTI was used to create 3D computational CED transport models and a semi-automatic segmentation scheme was used to assign the transport properties on a voxel by voxel basis [131]. The voxelized model approach is invaluable and advantageous because it is quicker than the tedious slice-by-slice segmentation [131].

## 4. Conclusions and Future Direction

This review paper discusses important investigations pertaining to the glymphatic system. The glymphatic system describes CSF flow within the perivascular spaces and extracellular spaces of the brain parenchyma with the help of AQP-4 on astrocytic end-feet for the interstitial waste solute drainage from the CNS. Nonetheless, some controversies and unresolved questions challenge glymphatic system studies and suggest alternative pathways for waste drainage out of the brain. One unresolved question is regarding the dominant solute transport mechanism for the glymphatic system. The roles of bulk flow and diffusion, as well as their driving forces, are still unsettled. Another unresolved question is whether glymphatic system dysfunction is the cause or the effect (or both) of numerous neurological diseases [34]. Various glymphatic studies have suggested that glymphatic dysfunction is responsible for the waste accumulation and may lead to various neurological diseases [132] including beta-amyloid and tau protein accumulation in Alzheimer’s disease, alpha-synuclein protein accumulation in Parkinson’s disease, and huntingtin accumulation in Huntington’s disease. The effect of AQP-4 on waste solute clearance [113] and the direction of glymphatic transport along the perivascular spaces [5,20,54,113,133,134] have also been debated. Quantitative measurements of the brain’s CSF flow and AQP-4 activity in vivo are required to completely understand the glymphatic system. Maintaining healthy sleep patterns and daily physical exercise may improve protein waste evacuation, perivascular fluid circulation, ISF-CSF exchange, and cognitive functioning with aging [135,136]. Sustaining meningeal lymphatic vessel functioning with aging might also improve CNS waste clearance and provide promising results for alleviating cognitive decline [29]. Significantly more work is required to address the current inconsistencies, and new experiments with distinct imaging techniques may help to analyze the development and prevention of neurodegenerative diseases.

In order to further our current understanding of the glymphatic system, future models and research should (1) include both diffusion and bulk flow terms into the modeling algorithm for an improved depiction of waste efflux to provide a more realistic fluid flow analysis; (2) determine the impact of various sedatives, sleep, and wakefulness on the bulk flow and diffusion measurements in the perivascular and extracellular spaces; (3) focus on systematically investigating the glymphatic system efflux pathways, which include the meningeal lymphatic pathway; and (4) develop therapeutic applications for the glymphatic system.
